# Nutritional Quality and Safety of Complementary Foods Developed from Blends of Staple Grains and Honey Bee Larvae (*Apis mellifera*)

**DOI:** 10.1155/2021/5581585

**Published:** 2021-05-10

**Authors:** Shewangzaw Addisu Mekuria, John N. Kinyuru, Beatrice Kiage Mokua, Mesfin Wogayehu Tenagashaw

**Affiliations:** ^1^Department of Food Science and Nutrition, Jomo Kenyatta University of Agriculture and Technology, P.O. Box 62000-00200, Nairobi, Kenya; ^2^University of Gondar, P.O. Box 196, Gondar, Ethiopia; ^3^Department of Applied Human Nutrition, Bahir Dar University, P.O. Box 26 Bahir Dar, Ethiopia

## Abstract

Complementary foods must be adequate to satisfy the nutritional needs of the growing child together with breastfeeding. This study was aimed at evaluating the nutritional composition, microbial safety, and sensory quality of extruded complementary foods developed from blends of staple grains and insect bee larva (*Apis mellifera*). Teff, maize, soybean, and bee larva samples were milled to flour and blended before extrusion as follows: ComF_01_ (57% maize, 29% teff, and 14% soybean) and ComF_02_ (58% maize, 29% teff, and 13% bee larvae) using NutriSurvey software (version, 2007). Nutrient composition, microbial, and sensory analyses of developed flour blends were conducted using standard methods. The proximate composition of moisture, fat, fiber, carbohydrate, and energy was significantly different between the developed and commercial wean-mix foods. ComF_02_ recorded the highest fat content (14.3 g/100 g), energy (427.18 kcal/100 g), and vitamins A (706 *μ*g/100 g), B3 (8.2 mg/100 g), and B9 (86.7 mg/100 g) while ComF_01_ had the highest protein content (12.56 g/100 g). Iron (40.94 mg/100 g) and calcium (68.20 mg/100 g) were the minerals with the highest content in ComF_02_. Both ComF_01_ and ComF_02_ met the recommended dietary allowance of nutrients for infants aged 6-12 months. Overall, the present study showed that bee larvae can be used to develop complementary foods that are nutritionally adequate, microbiologically safe, and sensory acceptable meeting the dietary allowance of infants at an acceptable level compared to conventional cereal-based foods.

## 1. Introduction

Complementary feeding is the process of providing alternative foods when breast milk alone is no longer sufficient to meet the nutritional requirements of infants, and therefore, other foods and liquids are needed, along with breast milk. Therefore, the infant transitions from exclusive breastfeeding to family foods. This period is typically from 6 to 24 months of age, even though breastfeeding may continue to two years of age and beyond [1]. This is a critical period of growth during which nutrient deficiencies and illnesses contribute globally to higher rates of undernutrition among children under five years of age [2].

While cereals are typically low in protein, cereal supplementation with local legumes that are high in protein improves the protein content of cereal-legume blends [3]. However, these plant diets are inadequate in terms of protein quality hence the need to include animal proteins [4]. Also, due to the increasing cost of animal proteins, food insecurity, population growth, and increasing need for protein-rich food [5], there should find another alternative.

Affordable animal proteins such as edible insects are a strong source of protein with the potential for use in CFs [6, 7]. Insects are protein sources as a nutritious novel food [8]. Insect protein processing is most similar to legume protein processing [9]. The use of edible insects in CF production is not studied abundantly. Only a few studies were conducted using insects as components of CFs such as grasshoppers [7], palm weevil larvae [10, 11], termites [12, 13], crickets [10], and *akokono* [14] with other local foods. However, a lack of data and information on insect supply and consumption [15] and culture, taboos, customs, and ethnic preferences have highly influenced the consumption of edible insects [16]. Among insects, honey bee larvae (*A. mellifera*) are a highly promising food resource since honey bees are reared worldwide and eaten as a delicacy in many cultures [17]. Therefore, CFs developed from locally available and acceptable food materials are possible and, when well-formulated, are appropriate for resource-poor settings [18]. Typically, food materials will include staple cereals or starchy tubers [19].

Commercial infant food is very costly in developing countries and may be unavailable to low-income families. Hence, the production of CFs on a small industrial scale may be less costly and affordable to the majority of the population. However, this must have an “easy-to-swallow” consistency and be microbiologically safe when consumed [20]. One of the common industrial techniques for processing CFs is extrusion cooking. This method has various beneficial effects such as reduction of antinutritional factors [21, 22], starch gelatinization [23], increased soluble dietary fiber [24, 25], decreased lipid oxidation [26], and decreased microorganism contamination [27] because of increased temperature and reduced moisture in the foods during extrusion [28]. Studies by [29] reported that teff-based CFs using extrusion cooking influenced nutrient compositions of the developed CFs. Children are at risk of exposure to food-borne pathogens with the introduction of CFs. Therefore, the microbial quality of the food is one of the most important criteria in terms of consumers' requirements for CFs [28]. Thus, appropriate safe and nutrient-dense CFs [30] should be provided at an appropriate age and development for child growth. This study, therefore, was aimed at evaluating the nutritional composition, microbial safety, and sensory acceptability of complementary foods developed from blends of staple grains and insect bee larva (*Apis mellifera*) using the extrusion cooking method.

## 2. Materials and Methods

### 2.1. Sample Collection and Preparation

#### 2.1.1. Sample Collection

The bee larva (*A. mellifera*) was aseptically collected from the University of Gondar apiary farm of modern beehives. The bees were reared in a suitable hygienic environment and fed natural pollen and nectar used for honey production. The fresh larva combs were immediately taken from the beehives and the larvae removed manually from each comb by swing and impact. Red teff (*Eragrostis tef* (*Zucc.*)), maize (*Zea mays L.*), and soybean (*Glycine max*) were purchased from the Gondar city local market and Gondar Agricultural Research Center, Ethiopia.

#### 2.1.2. Sample Preparation

The bee larvae were oven-dried at 60°C for 24 h, ground to powder, and packaged in an airtight polyethylene bag until analysis [17, 31]. Teff (*Eragrostis tef* (*Zucc. Trotter*)) grains were cleaned, washed with tap water until all undesirable components were removed, and then dried by sunlight and grounded to a fine flour using a local stone mill [29, 32]. Maize grain (*Zea mays L.*) was cleaned, soaked in potable water for 6 h, drained, germinated at room temperature for 48 h, and then sun-dried, dehulled, and milled in local mills to particle sizes ranging from 0.6 mm to 1.0 mm in diameter [33, 34]. The soybeans (*Glycine max*) were cleaned, boiled (for 30 min), dehulled, and dried at 60°C for about 13 h, milled into flour, and sieved (0.5 mm sieve) [29, 35]. Finally, the flour samples were packed in Ziplock polyethylene bags, labeled, and stored at room temperature until the extrusion process was carried out.

### 2.2. Formulation of Complementary Foods

**Figure 1:**
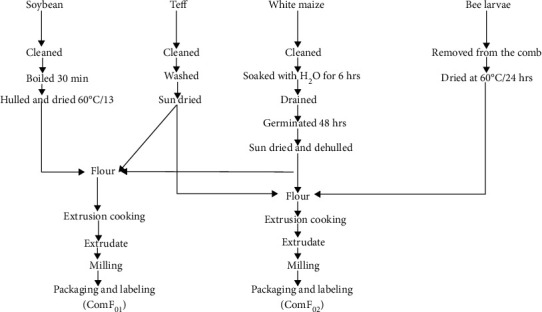
Flow chart of sample processing, formulation, and complementary food products.

Three composite flours with two different ratios were formulated (Figure 1) using NutriSurvey software (version, 2007) according to the guidelines of complementary feeding for children aged 6-12 months [2]. The first CF (ComF_01_) was composed of maize, teff, and soya bean flours in the ratio of 57 : 29 : 14, respectively. Similarly, the second CF (ComF_02_) consisted of maize, teff, and bee larva flours in the ratio of 58 : 29 : 13, respectively. Commercial (Enriched Mama's Choice) prepared infant food was bought from the market for comparison with the formulated flours.

### 2.3. Extrusion Processing

For ease of extrusion and best product quality, the following extrusion parameters were established for a blend formulation of composite flours, using a pilot-scale twin-screw extruder (model Clextral, BC-21 No. 124, Clextral, Firminy, France): moisture content (17%), barrel temperature (150°C), and screw speed [36] with a 29 g/min feed rate. A die plate was used with four circular holes, each with a whole 10 mm diameter. The extrusion feed and water flow rates were adjusted to determine the required moisture content of the samples and were calculated by using [37]

(1) $$\begin{array}{c} \text{Wa}=\text{Sw}*\frac{M-\text{Mo}}{100-M}, \end{array}$$

where Wa is the weight of water (g), Sw is the sample flour weight (g), Mo is the original flour moisture content (%), and *M* is the required moisture content (%).

The extrudates were finally dried and stored at ambient temperatures and then ground using a laboratory-scale mill (high-speed multifunctional grinder model-200) fitted with a 0.5 mm sieve. The flours produced from CFs were packaged in high-density polyethylene bags and stored at dry room temperature until laboratory analyses were carried out.

### 2.4. Nutrient Analysis

#### 2.4.1. Proximate Analysis of Raw Ingredient Flours and Extruded Complementary Foods

The proximate composition of the flours from raw ingredients, the developed complementary foods, and the commercial wean mix were analyzed according to the AOAC International standard methods [38]. Moisture content was determined by the drying method using hot-air oven circulation (method #925.09). Ash content of a known weight sample was determined through incineration (550°C) using a muffle furnace (method #923.03). Crude protein was determined by micro-Kjeldahl (method #979.09) and calculated by multiplying the corresponding total nitrogen content by a factor of 6.25. The crude fat content of the sample was determined by a Soxhlet extractor (method #930.09). Crude fiber content was determined by the following method #962.09. Available carbohydrate was calculated by the difference while energy was calculated using Atwater's calorie conversion factors of 4 kcal/g for crude protein, 9 kcal/g for crude fat, and 4 kcal/g for available carbohydrate [39].

#### 2.4.2. Micronutrient Analysis

The mineral content of CFs, namely, iron (Fe), zinc (Zn), and calcium (Ca), was determined using a flame atomic absorption spectrometry (Shimadzu AA-6200; Shimadzu, Tokyo, Japan) according to AOAC method 985.35 [38]. The *β*-carotene content was determined using column chromatography and a Shimadzu UV-Vis spectrophotometer (UV-1601PC, Japan) [40]. The results were converted to vitamin A values using the conversion factor of 6 *μ*g *β*-carotene: 1 *μ*g RE according to [41]. Vitamin B-complex (B1, B2, B3, B6, and B9) was determined by using High-Performance Liquid Chromatography (HPLC) (Shimadzu, RID-6A) [12].

#### 2.4.3. Antinutrient Analysis

Phytate content was determined according to [42] using the HPLC tannin, and saponin content was determined colorimetrically [43] with a UV-Vis spectrophotometer (Shimadzu model UV–1601 PC, Kyoto, Japan).

#### 2.4.4. Contribution of Complementary Foods to Recommended Dietary Allowance (RDA)

The average contribution of CFs to the RDA of each nutrient was calculated as a percent of the RDA [10].

(2) $$\begin{array}{c} \%\text{RDA}=\frac{\text{Amount of nutrient analyzed}}{\text{RDA for a given nutrient}}\times 100. \end{array}$$

#### 2.4.5. Bioavailability of Minerals

The molar ratios of phytate to zinc, calcium, and iron were calculated as the millimoles of phytate intake per day divided by the millimoles of zinc, calcium, and iron intake per day, respectively [44].

### 2.5. Microbial Analysis

Microbial analysis was conducted for each CF at day one, three months, and sixth months of storage and commercial wean mix. A serial dilution was done by tenfold and spread-plating techniques. A 1 g sample of each CFs was separately measured aseptically and dispensed separately and mixed with 10 ml diluent saline solution. Then, the food samples were homogenized in diluent for 1 minute using a vortex shaker (Cat AC-H311 made in India). Serial dilutions were made up to 10^10^ dilution factors [45]. From this homogenized food sample, 1 ml of the sample was transferred to the first cleaned and sterile test tube containing 9 ml diluent by sterile pipette to make a serial 10^−2^ and 10^−4^ dilutions of the homogenized sample. Each of these procedures was done in triplicate for each food sample. From each dilution, 1 ml of aliquot was transferred to a Petri dish and spread with a sterile bent glass rod on different types of solid media for the microbial count. To determine the developed foods of total plate count, *Escherichia coli* (*E. coli*), *Staphylococcus*, *Salmonella*, and *Shigella spp*, in the food plate count agar, eosin methylene agar, mannitol salt agar, Salmonella, and Shigella agar, respectively, were used and incubated for 24-36 h at 35°C [46, 47]. Yeast and molds were determined by spreading the aliquot on presolidified potato dextrose agar supplemented with 0.1 g chloramphenicol and incubated at 28°C for 5-7 days. Results of the counted colonies were reported as log_10_ CFU/g [48].

### 2.6. Sensory Analysis

A sensory evaluation for the acceptability of each CF gruel was done following the instructions of [2]. Gruel was made by adding 50 g of flour to 250 ml of water and cooked. Sensory analysis was performed using 30 semitrained mothers selected that had an acceptable and positive attitude towards eating the products from Gondar town, Ethiopia. Orientation was given for each panelist, how to code sample products for evaluation of appearance, aroma, taste, texture/mouthfeel, and overall acceptability. The mothers were also informed to rinse their mouth with clean water before proceeding to the next food testing. A five-point hedonic scale (5 = like very much, 4 = like moderately, 3 = neither like nor dislike, 2 = dislike moderately, and 1 = dislike very much) was used [35, 49].

### 2.7. Statistical Analysis

Results of nutritional composition, microbial, and sensory analysis of CFs were presented as means and standard deviation. One-way ANOVA and Least Significant Difference (LSD) tests were used to determine the differences among means between the CFs using SPSS for Windows Version, 23. The level of significant difference at *P* < 0.05 was considered.

## 3. Results and Discussion

### 3.1. Nutritional Composition of Ingredients

The proximate composition of foods may be of interest in the food industry for product development, quality control, or regulatory purposes [50]. The proximate composition of individual food ingredients is presented in Table 1. Moisture content of food ingredients ranged from 6.04 to 13.36 g/100 g with high moisture content being recorded for maize (13.36 g/100 g). High protein (50.50 g/100 g) was recorded on soybean followed by bee larvae (45.70 g/100 g), maize (9.79 g/100 g), and teff (9.79 g/100 g). The protein content of soybean reported by [29, 51] was 35.59 and 27 g/100 g, respectively, which was lower than the present study. There were high records of carbohydrate content of teff, followed by maize, soybean, and bee larvae which were 69.55, 72.44, 14.34, and 14.24 g/100 g, respectively.

The fat content of soybean was higher than the value reported by [29] and slightly similar to values reported by [51]. Similar findings were on protein and fat content of soaked and germinated maize grain flours reported by [52]. However, flours of teff carbohydrate content were slightly lower than the report of [29, 51]. The variation of all these may be due to genotype, soil fertility, water availability, temperature, and environmental conditions during grain development [53] and the method of processing [54].

Results of honey bee larvae of ash, protein, and fat values of the present study were different from the reports of [31] who reported lower 35.3 g/100 g protein and 14.5 g/100 g fat. Moreover, [55, 56] reported lower ash, protein, and fat content of insect bee larvae compared to our study. The variation may be due to the species of bee insect [57], season and climate [31], and the type of insect feed [58].

### 3.2. Proximate Composition of CFs and Commercial Wean Mix

Extruded weaning foods were made from a combination of cereals and legumes to produce the correct protein and energy content for growing children [59]. Information about food composition is necessary for the assessment of diet quality and the development and application of food-based dietary guidelines, providing a useful tool for the field of public health nutrition [60].  Table 2 shows the proximate (g/100 g) and mineral (mg/100 g) composition of extruded complementary foods and commercial wean mix and energy content (kcal/100 g).

Moisture, ash, protein, fat, fiber, carbohydrate, and energy (kcal) content of the developed CFs and commercial wean mix met the requirements of the Codex Alimentarius Commission [61]. Statistical analysis showed that there was a statistically significant difference (*P* = 0.037) of ash content between the foods and high records with ComF_01_ (2.09 g/100 g). There was a significant difference (*P* ≤ 0.001) between the moisture, fat, fiber, carbohydrate, and energy content of the foods. Moisture, ash, protein, fat, fiber, carbohydrate, and energy (kcal) content of the developed CFs and commercial wean mix met the requirements of the Codex Alimentarius Commission [61].

There was a significant difference (*P* ≤ 0.001) between the moisture, fat, fiber, carbohydrate, and energy content of the foods. The moisture content of the foods ranged from 2.46 to 5.72 g/100 g. The ComF_02_ had the highest moisture content (5.72 g/100 g) while the commercial wean mix had the least (2.46 g/100 g). Proteins are important in both quantity and quality, for the rapid growth and development of a child. The protein content of ComF_01_, ComF_02_, and commercial wean mix was 12.56, 11.75, and 10.78 g/100 g, respectively. The significantly higher protein content in ComF_01_ may be due to soybean in the formulation which had higher protein content than bee larvae. However, the values of ComF_01_ and ComF_02_ were significantly higher than the commercial wean mix. There was higher fiber (4.52 g/100 g) content on ComF_01_ followed by ComF_02_ (3.47 g/100 g) and commercial wean mix (2.82 g/100 g). The highest fat (14.3 g/100 g) and energy (427.18 g/100 g) content was observed in ComF_02,_ while the least fat (2.82 g/100 g) and energy (385.25 g/100 g) were observed in the commercial wean mix. Nutritionally, findings of the energy content of ComF_02_ were higher than the ComF_01_ and commercial wean mix might be due to the blends of bee larva in that are high in fat.

Micronutrient deficiency is a common public health problem in developing countries, especially for infants and children in the first two years of life [65]. Iron, zinc, and calcium are important minerals in the complementary feeding of infants and young children. There was a significant difference (*P* ≤ 0.001) in mineral iron and calcium content between the developed foods and the commercial wean mix (Table 2). High values of iron (40.94 mg/100 g) and calcium (68.20 mg/100 g) were recorded in ComF_02_ and commercial wean mix, respectively. Mineral iron and calcium composition of both developed CFs were higher than the recommended value [62]. This might be due to the blends of insect bee larvae in complementary foods, which are rich in mineral content [31]. However, the mineral content of zinc did not fulfill the recommended value in all foods. Conversely, the commercial wean-mix zinc (2.32 mg/100 g) was found to be significantly lower (*P* = 0.006) than both food ComF_01_ and ComF_02_.

### 3.3. Vitamin Composition of CFs and Commercial Wean Mix

Establishing precise daily requirements for vitamins is not easy, and there was considerable individual variation; however, achieving the reference nutrient intake (RNI) should be possible with a healthy balanced diet [66]. The vitamin composition of the developed CFs and commercial wean mix is indicated in Table 3. There was a significant difference (*P* ≤ 0.001) of vitamin composition between the developed CFs and commercial wean mix. Results of vitamin A showed that ComF_02_ and commercial wean mix fulfilled the recommended value [63]. Vitamin A content of commercial wean mix (2.08 mg/100 g) was higher than that of ComF_01_ (0.16 mg/100 g) and ComF_02_ (0.71 mg/100 g), respectively. Vitamin A content of ComF_01_ (0.16 mg/100 g) did not meet the recommended value for infants and children. Vitamins thiamine (0.81 mg/100 g) and riboflavin (0.70 mg/100 g) value of ComF01 had highest values, however, low records of pyridoxine (0.29 mg/100 g) and folate (51 *μ*g/100 g). Also, niacin (8.20 mg/100 g) and folate (86.7 *μ*g/100 g) in ComF_02_ were high but could not meet the recommended value of riboflavin.

### 3.4. Contribution to Recommended Dietary Allowance (RDA)

Table 4 summarizes the percentage contribution of macro- and micronutrients provided by complementary foods and commercial wean mix which met RDA for 6-12 months. The contribution of ComF_01_, ComF_02_, and commercial wean mix to the RDA of protein was the highest which was 114.18, 106.82, and 98%, respectively; however, with the exception of the energy contribution from ComFs_02_, the contribution of the two CFs to the RDA of energy for infants was less than 50%. All foods had the potential to contribute to the RDA of iron (52.64-365.18%) and zinc (73.33-97.33%); however, the contribution of calcium (12.22-26.23%) intake was very low. The vitamins' potential contribution of CFs to the RDA were highest except ComF_01_ for vitamin A (33.34%).

### 3.5. Antinutrient Composition of CFs

Antinutrients in complementary foods for children could have a negative impact on nutritional status [68]. Therefore, using extrusion significantly reduced the antinutritional factors of the formulated diets [22]. Antinutrient composition of developed CFs in mg/100 g is shown in Table 5.

Antinutritional (mg/100 g) content showed high records of tannins (208.93) and phytates (68.18) in ComF_01_ than in ComF_02_ and commercial wean mix which showed tannins of 119.37 and 63.60 and phytates of 13.13 and 10.46, respectively. However, saponins were not detected in both CFs and commercial wean mix. Cereal-based complementary foods are high in phytates which limit the bioavailability of nutrients, including iron, calcium, zinc, and, in some cases, proteins, which are crucial to the development of infants [69]. Tannin-protein complexes can contribute to digestive enzyme inactivation and decrease the digestibility of proteins by protein substrate association with ionizable iron [70]. The existence of tannins in food could reduce the quality of foods, suppress growth, reduce iron absorption, harm the gastrointestinal tract's mucosal lining, altercation excretion, and increase protein and critical amino acid excretion [22].

### 3.6. Bioavailability of Minerals

The calculated results of both CF concentration of phytate and its molar ratio of minerals calcium, iron, and zinc were in line with the recommended limits (Table 6).

The recommended limits of phytate to calcium, iron, and zinc were (phytate to calcium) < 0.24 for calcium [74], (phytate to iron) < 1 for iron [71], and (phytate to zinc) < 15 for zinc [72]. (0.14, 0.03, and 0.02), calcium (2.40, 0.45, and 0.45), and zinc (0.13, 0.02, and 0.01), respectively, showed good iron, zinc, and calcium bioavailability in both developed complementary foods and commercial wean mix. This might be due to the low amounts of phytate in the CFs. Also, the addition of legumes (ComF_01_) can slightly improve the iron content of those diets; however, the bioavailability of this nonheme iron source was lower than hemi iron [41]. According to the report of [75], lowering the phytic acid should enhance the bioavailability of iron and zinc in the extrudates as phytic acid has been implicated in making these minerals unavailable. Extrusion cooking results in the degradation of cereals and legumes' antinutrients by about 30% [76]. The inhibitory effect of phytate on bioavailability increases with the increment of phytate intake [77]. In the gastrointestinal tract, phytic acid binds trace elements and macro elements such as copper, calcium, magnesium, and iron to make dietary minerals inaccessible for consumption and used by the body [78].

### 3.7. Microbiological Loads of CFs

The microbial load of any food material is, however, a useful index of quality of the extrudate as well as revealing the potential safety status of the extruded food products from a human consumption point of view and storage of the products [45].  Table 7 summarizes the microbiological counts (log_10_ CFU/g) of the developed CFs at day one and after storage of three months and six months and commercial wean mix.

The microbial results of both CFs were below the acceptable level, i.e., acceptable limit (<5 log_10_ CFU/g) [64] and not detected on commercial wean mix. High levels of moisture (above 10%) exacerbate spoilage by encouraging microbial activity and chemical reactions that reduce the shelf life of the food [80]. Therefore, microbial counts of *E. coli*, *Staphylococcus*, *Salmonella*, and *Shigella spp*, total plate count, yeast, and mold of both CFs were within safe levels. This may be due to the quality control measures used during manufacturing and techniques [81, 82] as well as because of low moisture content [83].

On day one, microbial counts of both CFs were not detected. Similarly, at six months of storage, there were no *E. coli*, *Staphylococcus*, *Salmonella*, and *Shigella spp* detected. The findings of bacterial *Shigella*, *Salmonella*, and *Staphylococcus spp* of the present study were in line with the study of [84], which were not detected from the developed weaning food samples. This was likely since these were not persistent in the environment or were more likely destroyed during cooking [85, 86].

There was a slight increment of microbial load at three and six months of storage. The mean total plate count of ComF_01_ (3.36 log_10_ CFU/g) and ComF_02_ (3.04 log_10_ CFU/g) were recorded at three months and ComF_01_ (3.46 log_10_ CFU/g) and ComF_01_ (3.17 log_10_ CFU/g) at six months of storage. The detection of total plate counts in the CFs at an acceptable level could be due to cross-contamination through the migration of substances from the packaging into food [87] or storage time increased [45] or contamination of packaging material [88–90].

Yeast counts of ComF_01_ at three (2.0 log_10_ CFU/g) and six (2.30 log_10_ CFU/g) months of storage were as mean mold counts at three (2.17 log_10_ CFU/g) and six (2.60 log_10_ CFU/g) months of storage were recorded. Yeast counts of ComF_02_ at three (2.00 log_10_ CFU/g) and six months (2.18 log_10_ CFU/g) and mold at three (2.30 log_10_ CFU/g) and six months (2.40 log_10_ CFU/g) were recorded. The presence of molds and yeast development in the developed CFs after three and six months of storage might be due to packaging or storage conditions. Most yeasts and molds were obligatorily aerobic, and their temperature range (10-35°C) was also broad [91]. Comparing yeast and molds, higher mold counts (CFU/g) of the developed CFs were recorded than yeast in both CF_S_ but not detected on commercial wean mix. This may be due to molds' moisture requirements being relatively low, although yeasts generally require higher water activity [92]. Therefore, suppression of microbial growth appears to be favored under high temperature and low moisture environments [93].

### 3.8. Sensory Analysis

The sensory analysis relies on consumers to provide the data on which decisions were based [94]. Sensory attributes and overall acceptance of commercial wean mix (Table 8) were higher than those of ComF_01_ and ComF_02_. The small number of participants used in the sensory test increased the chance of getting accurate and reliable results [95]. There was a statistically significant difference (*P* = 0.003) in appearance and aroma between the developed CFs and commercial wean mix. The appearance was an important attribute in food choice and acceptance [96]. The outcome of the sensory evaluation indicated that ComF_01_ and ComF_02_ samples were similar in appearance while the commercial wean mix differed significantly. However, ComF_01_ had a lower score of overall acceptance than ComF_02_. This may be due to the higher presence of tannins in ComF_01_. According to the report of [75], tannins also decrease palatability. There was a highly significant difference (*P* ≤ 0.001) between the foods with regard to taste, texture, and overall acceptability. The aroma, taste, and overall acceptability of ComF_02_ were liked more by the panelists as compared to those of ComF_01_ but were of lower acceptability than those of the commercial wean mix. The best score rating of the commercial wean mix would be as a result of flavoring additions in the product [97].

Infants and toddlers present a challenge to sensory and consumer researchers because of their inability to communicate verbally, limited cognitive abilities, and very low attention span [98]. Besides, most found young children to have lower sensitivity than adults [99]. Sensory testing with infants and young children, therefore, has often employed indirect approaches. Therefore, the sensory evaluation of the present study was conducted by mothers or caretakers. For preference evaluation, parents' liking is important in deciding if a given CF would be suitable for their infants [100, 101]. All sensory evaluations of the developed CFs and commercial wean mix were above the minimum threshold i.e., the hedonic scale which was equal to three, neither likes nor dislikes [10].

## 4. Conclusions

The present study revealed that the development of CFs using extrusion cooking makes the products desirable in nutritional quality, microbial safety, and sensory acceptability. The nutritional composition of the developed foods meets the Codex Alimentarius Standard of the recommended dietary allowance for infants. The potential of using bee larvae as a novel ingredient in the development of CFs has been an alternative protein gained from plant-based protein sources. Using bee larvae with staple grains as a component of CFs is appropriate for macro- and micronutrient improvement of CF development for infants and young children. The extruded complementary foods containing bee larvae can be microbially safe and acceptable similar to cereal-based complementary foods and have the potential to contribute to the recommended dietary allowance. However, further study should be done on the effects of the developed CF foods on biochemical, hematological, and histopathological changes using laboratory animals.

## Figures and Tables

**Table 1:** Proximate composition of individual food ingredients (g/100 g, dried weight basis).

Ingredients	Moisture (%)	Ash	Protein	Fat	Fiber	Carbohydrate
Bee larvae	8.68 ± 0.17^b^	3.66 ± 0.19^b^	45.70 ± 0.85^b^	24.98 ± 0.12^a^	2.74 ± 0.10^c^	14.24 ± 0.59^c^
Soybean	6.04 ± 0.10^c^	5.04 ± 0.07^a^	50.50 ± 0.50^a^	19.53 ± 0.53^b^	4.54 ± 0.22^a^	14.34 ± 1.13^c^
Red teff	8.45 ± 0.05^b^	3.44 ± 0.04^b^	9.79 ± 0.07^c^	2.65 ± 0.06^d^	3.23 ± 0.10^b^	72.44 ± 0.06^a^
White maize	13.36 ± 0.21^a^	0.61 ± 0.19^c^	10.10 ± 0.13^c^	4.88 ± 0.21^c^	1.50 ± 0.06^d^	69.55 ± 0.12^b^

The results are presented as the SD of the means. Means with different superscripts (alphabets) in the same column are significantly different, *P* < 0.05.

**Table 2:** Proximate (g/100 g), energy content (kcal/100 g), and mineral (mg/100 g) composition of extruded complementary foods and commercial wean mix.

Nutrients	ComF^01^	ComF^02^	Commercial wean mix	*P* value	RV^+^
Moisture	4.41 ± 0.19^b^	5.72 ± 0.17^a^	2.46 ± 0.39^c^	<0.001	10*^α^*
Ash	2.09 ± 0.09^a^	1.88 ± 0.04^b^	2.01 ± 0.08^ab^	0.037	<4*^α^*
Protein	12.56 ± 0.17^a^	11.75 ± 0.15^b^	10.78 ± 0.29^c^	<0.01	15^+^
Fat	12.4 ± 0.1^b^	14.3 ± 0.1^a^	2.82 ± 0.36^c^	<0.001	10-25^+^
Fiber	4.52 ± 0.04^a^	3.47 ± 0.08^b^	2.75 ± 0.17^c^	<0.001	<5^+^
Carbohydrate	64.02 ± 0.41^b^	62.87 ± 0.23^c^	79.19 ± 0.55^a^	<0.001	60-75^∞^
Energy	417.93 ± 3.23^b^	427.18 ± 2.42^a^	385.25 ± 1.77^c^	<0.001	400-425^+^
Fe	40.17 ± 0.38^b^	40.94 ± 0.29^a^	5.79 ± 0.16^c^	<0.001	9.3^∗^
Zn	2.84 ± 0.18^a^	2.92 ± 0.16^a^	2.32 ± 0.11^b^	0.006	4.1^∗^
Ca	31.78 ± 0.11^c^	44.34 ± 0.49^b^	68.20 ± 0.12^a^	<0.001	0.40^∗^

The results are presented as SD of the means. ComF^01^: complementary food 01 (white maize+red teff+soybean); ComF^02^: complementary food 02 (white maize+red teff+insect bee larvae); RV: recommended value; commercial wean mix (Enriched Mama's Choice); means with different superscripts (alphabets) in the same row are significantly different (*P* < 0.05); ^∗^sources: [62, 63]; ^+^source [32]; *^α^*[64]; ^∞^estimated from data given for protein and fat in the codex standard.

**Table 3:** Vitamin composition of complementary foods and commercial wean mix per 100 g.

Vitamins	ComF_01_	ComF_02_	Commercial wean mix	*P* value	RV*^α^*
Vitamin A (*μ*g)	167.3 ± 5.84^c^	706.8 ± 16.28^b^	2082.02 ± 85.08^a^	<0.001	400
B1 (thiamine) (mg)	0.81 ± 0.09^a^	0.48 ± 0.06^b^	0.24 ± 0.36^c^	<0.001	0.36
B2 (riboflavin) (mg)	0.70 ± 0.07^a^	0.26 ± 0.03^c^	0.44 ± 0.20^b^	<0.001	0.36
B3 (niacin) (mg)	5.23 ± 0.41^bc^	8.20 ± 0.32^a^	6.33 ± 1.43^c^	<0.001	6.0
B6 (pyridoxine) (mg)	0.29 ± 0.019^c^	0.45 ± 0.01^ab^	0.53 ± 0.47^a^	<0.001	0.44
B9 (folate) (*μ*g)	51.0 ± 4.9^c^	86.70 ± 1.80^a^	77.76 ± 16.31^ab^	<0.001	83

The results are presented as SD of the means. ComF_01_: complementary food 01 (white maize+red teff+soybean); ComF_02_: complementary food 02 (white maize+red teff+insect bee larvae); RV: recommended value; commercial wean mix (Mama's Choice); means with different superscripts (alphabets) in the same row are significantly different *P* < 0.05; *^α^*sources [63].

**Table 4:** Percentage contribution of macro- and micronutrients provided by complementary foods and commercial wean mix meeting RDA for 6-12 months.

Nutrients	RDA	Percentage RDA met		
		ComF_01_	ComF_02_	Commercial wean mix
Energy (kcal)	850	49.17	50.26	45.32
Protein (g/day)	11	114.18	106.82	98
Carbohydrate (g)	95	67.39	66.19	83.36
Fat (g)	30	41.33	47.67	9.40
Ca (mg)	260	12.22	17.05	26.23
Zn (mg/day)	3	94.67	97.33	73.33
Fe (mg/day)	11	365.18	372.18	52.64
Vitamin A (*μ*g RE)	500	33.34	141.36	416
B1 (thiamine) (mg/day)	0.3	270.67	160	80
B2 (riboflavin) (mg/day)	0.4	175	65	110
B3 (niacin) (mg/day)	4.0	130.75	205	158.25
B6 (pyridoxine) (mg/day)	0.3	96.67	150	176.67
B9 (folate) (*μ*g/day)	80	63.75	108.38	97.2

ComF_01_: complementary food 01 (white maize+red teff+soybean); ComF_02_: complementary food 02 (white maize+red teff+insect bee larvae); commercial wean mix (Mama's Choice); *^β^*source dietary reference intake [2, 67].

**Table 5:** Antinutrient composition of developed complementary foods (mg/100 g).

CF_S_	Antinutrients		
	Tannins	Phytates	Saponins
ComF_01_	208.93 ± 0.04^a^	68.18 ± 4.15^a^	nd
ComF_02_	119.37 ± 0.31^b^	13.13 ± 0.63^b^	nd
Commercial wean mix	63.69 ± 0.34^c^	10.46 ± 0.5^b^	nd
*P* value	<0.001	<0.001	—

The results are presented as SD of the means. ComF_01_: complementary foods (white maize+red teff+soybean); ComF_02_: complementary foods (white maize+red teff+insect bee larvae); nd: not detected.

**Table 6:** Minerals : phytate molar ratio in developed complementary foods and commercial wean mix.

Sample	Phytate : iron	Phytate : zinc	Phytate : calcium
ComF_01_	0.14	2.40	0.13
ComF_02_	0.03	0.50	0.02
Commercial wean mix	0.02	0.45	0.01
Limits	<1*^α^*	<15*^β^*	<0.24^∗^

ComF_01_: complementary foods 01 (white maize+red teff+soybean); ComF_02_: complementary foods 02 (white maize+red teff+insect bee larvae); commercial wean mix (Mama's Choice); *^α^*sources: [71]; *^β^*source: [72]; ^∗^[73].

**Table 7:** Microbiological counts (log_10_ CFU/g) of the developed complementary foods after three and six months of storage and commercial wean mix.

Microorganisms	At 3 months		At 6 months		Commercial wean mix	Limit^∗^
	ComF_01_	ComF_02_	ComF_01_	ComF_02_		
*E. coli*	nd	nd	nd	nd	nd	<1
*S. aureus*	nd	nd	nd	nd	nd	<1
*Salmonella*	nd	nd	nd	nd	nd	0/25 g
Shigella	nd	nd	nd	nd	nd	0/25 g
Total plate count	3.36	3.04	3.46	3.17	Nil	<5
Yeast	2.00	2.00	2.30	2.18	Nil	<3
Molds	2.17	2.30	2.60	2.40	Nil	<3

Results are presented as means. CFU: colony forming units; nd: not detected; ^∗^[64, 79].

**Table 8:** Sensory analysis of the developed complementary foods compared to commercial wean mix.

Characteristic	ComF_01_	ComF_02_	Commercial wean mix	*P* value
Appearance	3.77 ± 0.82^c^	4.07 ± 0.69^bc^	4.41 ± 0.74^a^	0.003
Aroma	3.73 ± 0.91^c^	4.23 ± 0.63^ab^	4.40 ± 0.80^a^	0.003
Taste	3.57 ± 0.73^c^	4.43 ± 0.63^ab^	4.50 ± 0.68^a^	<0.001
Texture/mouth feel	3.77 ± 0.71^c^	3.97 ± 0.61^bc^	4.47 ± 0.51^a^	<0.001
Overall acceptability	3.63 ± 0.61^c^	4.20 ± 0.55^b^	4.63 ± 0.49^a^	<0.001

The results are presented as SD of the means. ComF_01_: complementary foods 01 (white maize+red teff+soybean); ComF_02_: complementary foods 02 (white maize+red teff+insect bee larvae); commercial wean mix (Mama's Choice); means with different superscripts (alphabets) in the same row are significantly different (*P* < 0.05).

## Data Availability

The data used and/or analyzed in the study are available from the corresponding author on reasonable request.
